# COVID-19 in pediatric nephrology centers in Turkey

**DOI:** 10.55730/1300-0144.5521

**Published:** 2022-07-24

**Authors:** Emre LEVENTOĞLU, Yeşim ÖZDEMİR ATİKEL, Hülya NALÇACIOĞLU, İsmail DURSUN, Hasan DURSUN, Zeynep YÜRÜK YILDIRIM, Nurdan YILDIZ, Gülşah KAYA AKSOY, Mehmet TAŞDEMİR, Mehtap ÇELAKIL, Beltinge DEMİRCİOĞLU KILIÇ, Şenay ZIRHLI SELÇUK, Nur CANPOLAT, Evrim KARGIN ÇAKICI, Sare Gülfem ÖZLÜ, Sebahat TÜLPAR, Selçuk YÜKSEL, Bahriye ATMIŞ, Serra SÜRMELİ DÖVEN, Sevgin TANER, Pelin ERTAN, Aslı KAVAZ, Meral TORUN BAYRAM, Mukaddes KALYONCU, Kaan GÜLLEROĞLU, Caner KABASAKAL, Belde KASAP DEMİR, Rümeysa Yasemin ÇİÇEK, Ilmay BİLGE, Osman DÖNMEZ, Aslıhan KARA, Önder YAVAŞCAN, Gül ÖZÇELİK, Deniz GEZGİN YILDIRIM, Muhammet Akif GÜLER, Ferah SÖNMEZ, Hakan POYRAZOĞLU, Sema AKMAN, Rezan TOPALOĞLU, Harika ALPAY, Sevcan A. BAKKALOĞLU

**Affiliations:** 1 Departments of Pediatric Nephrology and Rheumatology, Faculty of Medicine, Gazi University, Ankara, Turkey; 2 Department of Pediatric Nephrology, Eskişehir City Training and Research Hospital, Eskişehir, Turkey; 3 Department of Pediatric Nephrology, Faculty of Medicine, Ondokuz Mayıs University, Samsun, Turkey; 4 Department of Pediatric Nephrology, Faculty of Medicine, Erciyes University, Kayseri, Turkey; 5 Department of Pediatric Nephrology, Prof. Dr. Cemil Taşçıoğlu City Hospital, University of Health Sciences, İstanbul, Turkey; 6 Department of Pediatric Nephrology, İstanbul Faculty of Medicine, İstanbul University, İstanbul, Turkey; 7 Department of Pediatric Nephrology, İstanbul Pendik Education and Research Hospital, Marmara University, İstanbul, Turkey; 8 Department of Pediatric Nephrology, Faculty of Medicine, Akdeniz University, Antalya, Turkey; 9 Departments of Pediatric Nephrology and Rheumatology, Faculty of Medicine, Koç University, İstanbul, Turkey; 10 Department of Pediatric Nephrology, Hatay State Hospital, Hatay, Turkey; 11 Department of Pediatric Nephrology, Faculty of Medicine, Gaziantep University, Gaziantep, Turkey; 12 Department of Pediatric Nephrology, Turgut Özal Medical Center, İnönü University, Malatya, Turkey; 13 Department of Pediatric Nephrology, Cerrahpaşa Faculty of Medicine, İstanbul University, İstanbul, Turkey; 14 Department of Pediatric Nephrology, Dr. Sami Ulus Maternity and Child Health and Diseases Training and Research Hospital, University of Health Sciences, Ankara, Turkey; 15 Department of Pediatric Nephrology, Ankara City Training and Research Hospital, Ankara, Turkey; 16 Department of Pediatric Nephrology, İstanbul Bakırköy Dr. Sadi Konuk Research and Training Hospital, University of Health Sciences, İstanbul, Turkey; 17 Department of Pediatric Nephrology, Faculty of Medicine, Pamukkale University, Denizli, Turkey; 18 Department of Pediatric Nephrology, Faculty of Medicine, Çukurova University, Adana, Turkey; 19 Department of Pediatric Nephrology, Faculty of Medicine, Mersin University, Mersin, Turkey; 20 Department of Pediatric Nephrology, Adana City Training and Research Hospital, University of Health Sciences, Adana, Turkey; 21 Department of Pediatric Nephrology, Faculty of Medicine, Celal Bayar University, Manisa, Turkey; 22 Department of Pediatric Nephrology, Faculty of Medicine, Osmangazi University, Eskişehir, Turkey; 23 Department of Pediatric Nephrology, Faculty of Medicine, Dokuz Eylül University, İzmir, Turkey; 24 Departments of Pediatric Nephrology and Rheumatology, Faculty of Medicine, Karadeniz Technical University, Trabzon, Turkey; 25 Department of Pediatric Nephrology, Faculty of Medicine, Başkent University, Ankara, Turkey; 26 Department of Pediatric Nephrology, Faculty of Medicine, Ege University, İzmir, Turkey; 27 Departments of Pediatric Nephrology and Rheumatology, Faculty of Medicine, İzmir Katip Çelebi University, İzmir, Turkey; 28 Department of Pediatric Nephrology, Başakşehir Çam and Sakura City Hospital, İstanbul, Turkey; 29 Departments of Pediatric Nephrology and Rheumatology, Faculty of Medicine, Uludağ University, Bursa, Turkey; 30 Departments of Pediatric Nephrology and Rheumatology, Faculty of Medicine, Fırat University, Elazığ, Turkey; 31 Department of Pediatric Nephrology, Faculty of Medicine, Medipol University, İstanbul, Turkey; 32 Department of Pediatric Nephrology, Şisli Hamidiye Etfal Training and Research Hospital, University of Health Sciences, İstanbul, Turkey; 33 Department of Pediatric Nephrology, Faculty of Medicine, Atatürk University, Erzurum, Turkey; 34 Department of Pediatric Nephrology, Faculty of Medicine, Adnan Menderes University, Aydın, Turkey; 35 Department of Pediatric Nephrology, Faculty of Medicine, Hacettepe University, Ankara, Turkey

**Keywords:** Children, COVID-19, kidney, pediatric nephrology, Turkey

## Abstract

**Background/aim:**

There is limited data on COVID-19 disease in children with kidney disease. We aimed to investigate the characteristics and prognosis of COVID-19 in pediatric nephrology patients in Turkey.

**Materials and methods:**

This was a national, multicenter, retrospective cohort study based on an online survey evaluating the data between 11^th^ March 2020 and 11^th^ March 2021 as an initial step of a detailed pediatric nephrology COVID-19 registry.

**Results:**

Two hundred and three patients (89 girls and 114 boys) were diagnosed with COVID-19. One-third of these patients (36.9%) were between 10–15 years old. Half of the patients were on kidney replacement therapy: kidney transplant (KTx) recipients (n = 56, 27.5%), patients receiving chronic hemodialysis (n = 33, 16.3%) and those on peritoneal dialysis (PD) (n = 18, 8.9%). Fifty-four (26.6%) children were asymptomatic. Eighty-two (40.3%) patients were hospitalized and 23 (28%) needed intensive care unit admission. Fifty-five percent of the patients were not treated, while the remaining was given favipiravir (20.7%), steroid (16.3%), and hydroxychloroquine (11.3%). Acute kidney injury developed in 19.5% of hospitalized patients. Five (2.4%) had MIS-C. Eighty-three percent of the patients were discharged without any apparent sequelae, while 7 (3.4%) died. One hundred and eight health care staff were infected during the study period.

**Conclusion:**

COVID-19 was most commonly seen in patients who underwent KTx and received HD. The combined immunosuppressive therapy and frequent exposure to the hospital setting may increase these patients’ susceptibility. Staff infections before vaccination era were alarming, various precautions should be taken for infection control, particularly optimal vaccination coverage.

## 1. Introduction

Coronavirus disease 2019 (COVID-19) is a novel viral disease caused by the Severe Acute Respiratory Syndrome Coronavirus 2 (SARS-CoV-2) virus, which has been declared a pandemic due to its global spread [1,2]. One year after the first confirmed case of COVID-19 in Turkey on March 11^th^, 2020, the total number of infected individuals reached approximately 3 million at the end of the first year [3]. The epidemiological characteristics of children infected with SARS-CoV-2 are limited; however, its incidence is much lower than in adults. Published studies in the early months of the pandemic suggest that children corresponded to less than 5% of all COVID-19 cases and they were less likely to become severely ill compared to adults [4,5]. However, with the spread of new variants, the frequency of pediatric patients has increased as of May 2021. Despite excellent overall pediatric outcomes, severe and fatal cases do occur [6]. Children with severe COVID-19 are more likely to have preexisting chronic medical conditions (e.g., cardiac, respiratory, neurodevelopmental, malignancy, or immunosuppression) [4,7–10]. However, most children present clinical symptoms ranging from asymptomatic to mild/moderate illness and relatively fewer children with COVID-19 require hospitalization or admission to the intensive care unit (ICU) [11].

Several reports regarding adults have reported underlying pathologies or preexisting comorbidities as important risk factors for severe COVID-19 [12]. Chronic kidney disease (CKD) was found to be associated with the severity and mortality of COVID-19 [13,14]. Specifically, patients with CKD had a three-fold risk of developing severe COVID-19 [15]. Also, pediatric patients on dialysis are at significant risk for experiencing infectious diseases such as COVID-19 because of their compromised immune system and their frequent exposure to the hospital setting [16]. About one-third of end-stage kidney disease (ESKD) patients with dialysis who were hospitalized with COVID-19 died [17]. The high mortality rate might be associated with the diminished immune system from uremia in ESKD patients [18]. However, pediatric nephrology (PN) patients, i.e. CKD, hemodialysis (HD), and kidney transplantation (KTx) patients and those on immunosuppressive treatment were reported to have a lower risk compared to adults in limited studies [5, 19–21].

We conducted this multicenter, national collaborative study to investigate the one-year data of characteristics and prognosis of COVID-19 in pediatric nephrology patients in Turkey as an initial step of a detailed pediatric nephrology COVID-19 registry.

## 2. Materials and methods

### 2.1. Study design

This was a nationwide, multi-center, retrospective cohort study based on the data collected from children and adolescents with COVID-19 who were followed up in pediatric nephrology departments between 11^th^ March 2020 (the day of detection of the first case in Turkey) and 11^th^ March 2021. An online questionnaire was sent to all pediatric nephrology centers in Turkey. The centers were requested to submit all confirmed cases of COVID-19 (inpatients and outpatients) under the age of 20 years. A total of 35 pediatric nephrology centers reported their COVID-19 cases during a study period.

The online questionnaire forms included information on demographics, primary renal diagnosis as well as kidney replacement therapy modality and associated medications, comorbid conditions such as obesity, hypertension, cardiovascular diseases or growth retardation and disease presentation, diagnostic tools for COVID-19, hospital and/or intensive care unit (ICU) stay, and outcome of the COVID-19 disease course.

This study was approved by the Ethics and Clinical Research Committee of Gazi University.

### 2.2. Diagnostic tools for COVID-19 and definitions

COVID-19 was diagnosed by laboratory confirmation using the reverse transcriptase-polymerase chain reaction (PCR) by Bio-Speedy® SARSCoV-2 real time PCR kit or serology tests. Also, patients with thorax tomography findings and those having highly suggestive clinical symptoms compatible with COVID-19 such as fever, cough, shortness of breath, tiredness, loss of taste and smell, headache, or myalgia were also diagnosed as COVID-19 [1]. COVID-19 disease severity, method of diagnosis, treatment, length of hospital stay, and disease outcome was documented. The severity of the disease was classified as mild, moderate, severe, or critical illness. The mild disease was defined as upper respiratory symptoms for a short duration or asymptomatic infection; moderate disease as mild pneumonia symptoms; severe disease as mild or moderate clinical features, plus any manifestations that suggest disease progression; critical illness as rapid disease progression, plus any other conditions like a respiratory failure with need for mechanical ventilation, septic shock, organ failure that needs monitoring in the intensive care unit [22].

Regarding comorbid conditions, obesity was defined as a body mass index above the 95^th^ percentile according to the national pediatric growth percentiles [23]. Hypertension was defined as an office blood pressure greater than the 95_th_ percentile according to age, sex, and height specific normative values in the Fourth Report [24]. The number of patients with the multisystem inflammatory syndrome (MIS-C), characterized by fever, systemic inflammation, and multiple organ involvement requiring hospitalization due to confirmed SARS-CoV-2 infection, was also reported [25]. Acute kidney injury was defined as an increase of serum creatinine by 0.3 mg/dL or 50% from baseline within 7 days, according to the Kidney Disease: Improving Global Outcomes [26].

### 2.3. Statistical analysis

In the presentation of descriptive statistics, the categorical data were expressed as numbers (percentages).

## 3.Results

Data were collected from a total of 35 centers, including 26 (74.3%) university hospitals and 9 (25.7%) Ministry of Health Affiliated Hospitals. Twenty-eight (77.1%) centers have HD, PD, and KTx facilities. Demographic and clinical characteristics of patients are given in Tables 1 and 2.

Among 203 patients (89 girls and 114 boys) diagnosed with COVID-19, one-third (36.9%) were between 10–15 years old and almost two-thirds (60.5%) were between 10–18 years. Only 5.4% of the patients were under 2 years of age (Table 1). Kidney transplant recipients comprised the main group (n = 56, 27.5%) and followed by patients receiving chronic HD (n = 33, 16.3 %), glomerulopathies (n = 31, 15.2%) and those on PD (n = 18, 8.9 %). Half of the patients with glomerulopathies were on a single or combination of conventional and/or biologic immunosuppressives. Other diagnostic groups are listed in Table 1. Almost half (n = 100, 49.3%) of the children had at least one comorbid condition, and 30% of them had multiple comorbidities.

Fifty-six percent of the patients were diagnosed when they applied to the hospital with complaints, either in the same clinic (n = 56, 42.3%) or in another health center (n = 28, 13.7%). Other patients had their diagnosis during PCR screening tests; 62 (30.5%) patients with a history of household contact with an index case were diagnosed via filiation, 18 (8.8%) patients with a routine PCR test before hospitalization for another reason. One hundred and seventy (83.7%) patients were diagnosed as COVID-19 disease by SARS-CoV-2 PCR test positivity, 12 (5.9%) by SARS-CoV-2 immunoglobulin M positivity, 11 (5.4%) by thorax tomography findings compatible with COVID-19 pneumonia and 10 (4.9%) by highly suggestive clinic symptoms like fever, cough or shortness of breath that were not explained by another cause.

Fifty-four (26.6%) children were asymptomatic, and 67 (33%) children presented with mild symptoms. Eighty-two (40.3%) patients were hospitalized and 23 (28%) of them needed ICU stay. The patients were most frequently hospitalized for 3–7 days (35.4%) and 7–14 days (31.7%). Only seven (8.5%) patients required >30 days of hospital stay. More than half of the patients (55%) were not treated, while the remaining patients were mostly given favipiravir (20.7%), steroids (16.3%), and hydroxychloroquine (11.3%). Some of the patients were treated with additional combination regimens of intravenous immunoglobulin (IVIG), low molecular weight heparin (LMWH), or biologic anticytokine therapies. Acute kidney injury developed in 19.5% of the hospitalized patients during the course of the disease. Five patients (2.4%) had the diagnosis of MIS-C. A total of 7 (3.4%) patients died during the study period. Of the patients who died, 3 (42.8%) died due to MIS-C, 2 (28.5%) due to COVID-19 pneumonia, 1 (14.2%) due to post-COVID cardiac arrest after discharge from the hospital, and 1 (14.2%) due to COVID-unrelated reason. Fortunately, 83% of the patients were discharged without any apparent sequelae.

In addition, a total of 108 healthcare staff members, 25 of whom were pediatric nephrologists and 27 of whom were nurses, were infected. Vaccination was available for healthcare staff in the second half of January 2021 and staff infections were all recorded prior to the vaccination era.

## 4. Discussion

In this one-year retrospective study, starting from the day of the first COVID-19 case detected in Turkey, the characteristics of 203 pediatric nephrology patients diagnosed with COVID-19 were evaluated. More than half of the patients were asymptomatic or mildly symptomatic. Although the hospitalization rate in PN patients was 40.3%, most of the patients were discharged without any sequelae. However, viral transmission to healthcare personnel in prevaccination era was remarkable.

COVID-19 disease is not only a respiratory infection but also a systemic disease that affects multiple organs. Since the SARS CoV-2 virus uses the angiotensin-converting enzyme-2 (ACE2) and transmembrane protease serine type 2 receptors to enter the cell and these molecules are highly expressed in kidney cells, the kidneys are one of the organs most frequently affected by COVID-19 disease. It causes directly parenchymal infection in tubule epithelial cells and podocytes, acute tubular injury, and acute tubular necrosis. The most common signs of renal involvement are increased serum creatinine level, proteinuria, and hematuria. Patients with preexisting kidney disease are at higher risk of developing serious illnesses from COVID-19 [27]. Therefore, it is essential to prevent kidney damage due to COVID-19 and to protect patients with kidney disease from the increased morbidity and mortality risks of COVID-19.

A study evaluating previously healthy patients in the 0–15 age group observed that 18.1% of patients were <1 year of age, 23.4% were 1–5 years, 33.9% were 6–10 years, 24.6% were 11–15 years [1]. In our study, when patients in the 0–15 age range with a known kidney pathology were evaluated, only 3.5% of patients were <1 year of age, 16.9% were 1–5 years, 26.7% were 6–10 years, 52.8% were 11–15 years. The reason why the frequency of COVID-19 in the first year of life was less compared to the literature is that our cases have various renal diseases, and the frequency of these pathologies increases with age.

In a study in which pediatric patients followed up with the diagnosis of inflammatory rheumatoid disease were compared with a healthy control group, it was shown that the presence of inflammatory rheumatoid disease increased the risk of symptomatic infection and hospitalization in terms of COVID-19 disease. It was also shown in that study that the risk of symptomatic infection increases with age [28]. In terms of kidney diseases, CKD was reported to be associated with increased risk, severity, and mortality of COVID-19 disease in adults, however, there is limited information on PN patients [15–17]. In a pediatric study from Spain, 18.7% of patients with COVID-19 had a KTx, and 18.7% were receiving chronic HD [29]. According to our data, 27.5% of the infected patients were KTx recipients, 16.3% were undergoing HD, 8.9% were on PD, 15.2% had glomerulopathies and 9.8% had nondialysis dependent CKD (stage I–V). The higher rates in PN patient groups may be due to their compromised immune system and their more frequent exposure to the hospital setting, i.e. in center HD patients. There is limited evidence in children with KTx suggesting not an increased risk of severe COVID-19 disease [30–33]. Evidence from similar adult KTx populations suggests an increased risk of acquiring COVID-19 and an increased risk of severe disease [34–39]. The incidence of COVID-19 was reported to be similar between pediatric KTx recipients and the general pediatric population [30]. In a study from Turkey evaluating 496 pediatric dialysis and KTx patients, the diagnosis of COVID-19 was made in 46 (9.2%) patients; among them, 63% were KTx recipients and 37% were on dialysis. Although the patients were usually asymptomatic or mildly symptomatic, 22 (47.8%) patients were hospitalized. One patient who underwent HD and had serious comorbid diseases died. The mortality rate was 2% in SARS-CoV-2 positive patients and 4.5% among hospitalized ones [40]. In our study, 82 (40.3%) patients were hospitalized for COVID-19. Twenty-three (11.3%) patients were followed up in the ICU. A total of 7 patients died, including 3 patients with MIS-C, 2 with COVID-19 pneumonia, 1 with sudden cardiac arrest at home after discharge, and 1 with COVID-19-unrelated reasons. We observed the hospitalization rate as 40.3% and the mortality rate as 3.4%. These rates are broadly similar in both studies.

In a study from Turkey including 76 pediatric patients without kidney disease who developed MIS-C, 35.5% of the patients were followed in the ICU and one patient died [41]. In another study conducted on 113 children with kidney disease, no MIS-C cases were reported [42]. Similarly, among pediatric dialysis and KTx patients, only two (0.4%) MIS-C cases were detected without any mortality [40]. Discrepant results in small studies may be due to baseline characteristics and comorbid conditions of the patients and highlighted the necessity of large studies to evaluate the role of underlying kidney disease as risk factors for MIS-C.

Unlike adults, most children present clinical symptoms ranging from asymptomatic to mild/moderate illness [5,43–46]. According to our observation, 26.6% of cases were asymptomatic and 33% cases were mildly symptomatic. Similarly, in another study evaluating children with chronic renal pathologies, 18.7% of patients were asymptomatic, and there were fever and/or cough as the symptoms in 50% of patients [29]. This may be related to the differences in pediatric innate immune system compared to adults, being familiar with other coronaviruses, having fewer ACE2 receptors in the nasal mucosa, or having fewer comorbidities such as diabetes and hypertension [44].

Acute kidney injury (AKI) is commonly seen during COVID-19 course, especially in patients with KTx [15,16,45]. Several systematic reviews reported the association of AKI in severe COVID-19 patients with a poorer prognosis. During COVID-19 course, 27% to 52% of adult KTx recipients and 36% of pediatric KTx recipients developed AKI [47–50]. In our study, 19.5% of hospitalized patients developed AKI. The lower rate of AKI in our study may be explained by recruited patient population which included not only transplant or stage 3–5 CKD patients, but also other kidney pathologies with normal basal kidney functions.

There is currently limited evidence to support the efficacy of a specific antiviral and/or immunomodulatory agent for the treatment of COVID-19 in adults, and no evidence in children [51–55]. In Turkey, antiviral therapy has been prescribed according to guidelines published by the Turkish Ministry of Health [56]. In the first months of the pandemic, hydroxychloroquine was used in the treatment of COVID-19 because it was observed that the risk of ICU admissions was decreased with the use of it [57]. However, since it was seen that it did not reduce the risk of hospitalization or mortality rates in later studies, its use in treatment was terminated [58]. In this study, we observed that treatment regimens were highly heterogeneous. Asymptomatic or mildly symptomatic patients were not treated while the remaining patients were mostly given favipiravir and steroids. Some of the patients were treated with additional combination regimens of IVIG, LMWH, or biologic anticytokine therapies. When our special patient group is taken into consideration, it should be kept in mind that drug interactions may occur due to the usage of multiple drugs, and the drug doses should be adjusted based on renal function. Additionally, there are no general rules about the management of immunosuppression. Despite an earlier hesitation on continuation/dose reduction of immunosuppression, studies in PN patients suggested that it should be maintained but maybe individually tailored based on disease severity, which was confirmed by an adult expert opinion in KTx [24,59]. In some studies, antimetabolites were typically reduced or discontinued, but calcineurin inhibitors were maintained if the clinic was not severe [24].

We observed that there were 108 infected healthcare staff, accounting for more than half of the COVID-19 positive patients. In a previous study, 204 (4.3%) healthcare staff were found to be positive for COVID-19 among a total of 4073 hospital staff. Attention was drawn to the importance of strict infection control measures to reduce viral transmission, as healthcare workers are at high risk for COVID-19 [60]. However, all these infections were recorded before the vaccination era in health care providers. The optimization of vaccination coverage should be highlighted to prevent subsequent infections.

In conclusion, among PN patients, COVID-19 was most commonly seen in patients who underwent KTx and undergoing HD. The combined immunosuppressive therapy and frequent exposure to the hospital setting may increase these patients’ susceptibility. In the study presenting data for the prevaccination period, a mortality rate of 3.4% and staff infections deserve more attention and further strict precautions. We acknowledge that the data on COVID-19 are rapidly evolving, and further studies are needed in pediatric kidney patients.

## Figures and Tables

**Table 1:** Demographic characteristics of patients with COVID-19.

**All patients with COVID-19, n (%)**	203 (100)	
**Age (years), n (%)**	0–1	5 (2.4)
	>1–2	6 (2.9)
	>2–5	18 (8.8)
	>5–10	38 (18.7)
	>10–15	75 (36.9)
	>15–18	48 (23.6)
	>18	13 (6.4)
**Gender, n (%)**	Female	89 (43.8)
**Patient groups according to renal replacement therapy or primary diagnostic category, n (%)**	Renal transplantation	56 (27.5)
	Hemodialysis	33 (16.2)
	Peritoneal dialysis	18 (8.8)
	ESKD	20 (9.8)
	Glomerulopathy	31 (15.2)
	Hypertension	10 (4.9)
	CAKUT	13 (6.4)
	Tubular diseases	8 (3.9)
	Cystic kidney diseases	4 (1.9)
	Other	10 (4.9)
**Comorbidities, n (%)**	Any comorbid condition	100 (49.3)
	Single comorbidity	70 (34.5)
	Multiple comorbidity	30 (14.8)
	Obesity	15 (7.4)

COVID-19: Coronovirus-19 disease; ESKD: End-stage kidney disease; CAKUT: Congenital anomalies of the kidney and urinary tract

**Table 2:** Clinical characteristics of patients with COVID-19.

**All patients with COVID-19, n (%)**	203 (100)	
**Outpatient clinic, n (%)**	Asymptomatic	54 (26.6)
	Mild symptomatic	67 (33)
**Hospitalized patients, n (%)**	Moderate	59 (29)
	Severe/critical	23 (11.3)
**How to reach a diagnosis, n (%)**	Filiation	62 (30.5)
	Admitting to hospital with complaints	86 (42.3)
	Routine PCR test prior to hospitalization for another reason	18 (8.8)
	Diagnosis at another center	28 (13.7)
	During a regular visit, screening tests due to family history	9 (4.4)
**Primary method for diagnosing COVID-19, n (%)**	SARS CoV-2 PCR test	170 (83.7)
	SARS CoV-2 IgM-IgG	12 (5.9)
	Thorax computer tomography	11 (5.4)
	High clinical suspicion	10 (4.9)
**Treatments, n (%)**	No treatment	112 (55)
	Favipiravir	42 (20.7)
	Oseltamivir	5 (2.5)
	Lopinavir-ritonavir	5 (2.5)
	Remdesivir	1 (0.5)
	Antibiotics	18 (8.9)
	Hydroxychloroquine	23 (11.3)
	Steroids	33 (16.3)
	Anakinra	6 (3)
	LMWH	8 (3.9)
	Aspirin	5 (2.5)
	IVIG	13 (6.4)
	Plasmaexchange	2 (1)
**Length of hospital stay, n (%) (n = 82)**	1–3 days	2 (2.4)
	3–7 days	29 (35.4)
	7–14 days	26 (31.7)
	14–21 days	14 (17)
	21–30 days	4 (4.8)
	30 days	7 (8.5)
**Outcomes, n (%)**	Acute kidney injury	16 (7.8)
	Patients discharged from ward with no sequelae	56 (27.6)
	Patients discharged from ward with sequelae	1 (0.5)
	Patients currently hospitalized in ward	2 (1)
	Patients discharged from ICU with no sequelae	12 (5.9)
	Patients discharged from ICU with sequelae	3 (1.5)
	Patients currently in ICU	1 (0.5)
	New oncet MIS-C and Kawasaki-like MIS-C	5 (2.5)
	Exitus	7 (3.4)

COVID-19: Coronavirus-19 disease; PCR: Polymerase chain reaction; SARS-CoV-2: Severe Acute Respiratory Syndrome Coronavirus 2; IgM: Immunoglobulin M; IgG: Immunoglobulin G; LMWH: Low molecular weight heparin; IVIG: Intravenous immunoglobulin; ICU: Intensive care unit; MIS-C: Multisystem inflammatory syndrome-children

## References

[1] Lu X Zhang L Du H Zhang J Li YY SARS-CoV-2 infection in children The New England Journal of Medicine 2020 382 17 1663 1665 10.1056/NEJMc2005073 32187458 PMC7121177

[2] Novelli G Biancolella M Mehrian-Shai R Erickson C Godri Pollitt KJ COVID-19 update: the first 6 months of the pandemic Human Genomics 2020 14 1 48 10.1186/s40246-020-00298-w 33357238 PMC7757844

[3] Bulut C Kato Y Epidemiology of COVID-19: What changed in one year? Turkish Journal of Medical Sciences 2021 51 SI-1 3253 3261 10.3906/sag-2110-153 34844297 PMC8771048

[4] CDC COVID-19 Response Team Coronavirus disease 2019 in children - United States, February 12–April 2, 2020 Morbidity and Mortality Weekly Report 2020 69 14 422 426 10.15585/mmwr.mm6914e4 32271728 PMC7147903

[5] Ludvigsson JF Systematic review of COVID-19 in children shows milder cases and a better prognosis than adults Acta Paediatrica 2020 109 6 1088 1095 10.1111/apa.15270 32202343 PMC7228328

[6] Mehta NS Mytton OT Mullins EWS Fowler TA Falconer CL SARS-CoV-2 (COVID-19): What do we know about children? A systematic review Clinical Infectious Diseases 2020 71 9 2469 2479 10.1093/cid/ciaa556 32392337 PMC7239259

[7] Shekerdemian LS Mahmood NR Wolfe KK Riggs BJ Ross CE Characteristics and outcomes of children with coronavirus disease 2019 (COVID-19) infection admitted to US and Canadian pediatric intensive care units JAMA Pediatrics 2020 174 9 868 873 10.1001/jamapediatrics.2020.1948 32392288 PMC7489842

[8] Tagarro A Epalza C Santos M Sanz-Santaeufemia FJ Otheo E Screening and severity of coronavirus disease 2019 (COVID-19) in children in Madrid, Spain JAMA Pediatrics 2020 8 e201346 10.1001/jamapediatrics.2020.1346 PMC7142799 32267485

[9] Parri N Lenge M Buonsenso D Coronavirus Infection in Pediatric Emergency Departments (CONFIDENCE) Research Group Children with Covid-19 in Pediatric Emergency Departments in Italy The New England Journal of Medicine 2020 383 2 187 190 10.1056/NEJMc2007617 32356945 PMC7206930

[10] DeBiasi RL Song X Delaney M Bell M Smith K Severe coronavirus disease-2019 in children and young adults in the Washington, DC, Metropolitan Region The Journal of Pediatrics 2020 223 199 203 10.1016/j.jpeds.2020.05.007 32405091 PMC7217783

[11] Doná D Torres Canizales J Benetti E Cananzi M De Corti F Pediatric transplantation in Europe during the COVID-19 pandemic: Early impact on activity and healthcare Clinical Transplantation 2020 34 10 e14063 10.1111/ctr.14063 32786120 PMC7435500

[12] Wang D Hu B Hu C Zhu F Liu X Clinical characteristics of 138 hospitalized patients with 2019 novel coronavirus-infected pneumonia in Wuhan, China JAMA 2021 323 11 1061 1069 10.1001/jama.2020.1585 PMC7042881 32031570

[13] Cheng Y Luo R Wang K Zhang M Wang Z Kidney disease is associated with in-hospital death of patients with COVID-19 Kidney International 2020 97 5 829 838 10.1016/j.kint.2020.03.005 32247631 PMC7110296

[14] Portolés J Marques M López-Sánchez P de Valdenebro M Muñez E Chronic kidney disease and acute kidney injury in the COVID-19 Spanish outbreak Nephrology Dialysis Transplantation 2020 35 8 1353 1361 10.1093/ndt/gfaa189 PMC7462722 32871592

[15] Henry BM Lippi G Chronic kidney disease is associated with severe coronavirus disease 2019 (COVID-19) infection International Urology and Nephrology 2020 52 6 1193 1194 10.1007/s11255-020-02451-9 32222883 PMC7103107

[16] Shen Q Wang M Che R Li Q Zhou J Consensus recommendations for the care of children receiving chronic dialysis in association with the COVID-19 epidemic Pediatric Nephrology 2020 35 7 1351 1357 10.1007/s00467-020-04555-x 32333285 PMC7181108

[17] Valeri AM Robbins-Juarez SY Stevens JS Ahn W Rao MK Presentation and outcomes of patients with ESKD and COVID-19 Journal of the American Society of Nephrology 2020 31 7 1409 1415 10.1681/ASN.2020040470 32467113 PMC7350989

[18] Betjes MG Immune cell dysfunction and inflammation in end-stage renal disease Nature Reviews Nephrology 2013 9 5 255 265 10.1038/nrneph.2013.44 23507826

[19] Kalra S Prevention and control of COVID-19 in chronic kidney disease: Correspondence Indian Journal of Pediatrics 2020 87 11 968 969 10.1007/s12098-020-03398-6 32638335 PMC7338340

[20] Gandolfini I Delsante M Fiaccadori E Zaza G Manenti L COVID-19 in kidney transplant recipients American Journal of Transplantation 2020 20 7 1941 1943 10.1111/ajt.15891 32233067 PMC7228396

[21] Marlais M Wlodkowski T Al-Akash S Ananin P Bandi VK COVID-19 in children treated with immunosuppressive medication for kidney diseases Archives of Disease in Childhood 2020 106 8 798 801 10.1136/archdischild-2020-320616 33355203 PMC7754669

[22] Qiu H Wu J Hong L Luo Y Song Q Clinical and epidemiological features of 36 children with coronavirus disease 2019 (COVID-19) in Zhejiang, China: an observational cohort study The Lancet Infectious Diseases 2020 20 6 689 696 10.1016/S1473-3099(20)30198-5 32220650 PMC7158906

[23] Neyzi O Bundak R Gökçay G Günöz H Furman A Reference values for weight, height, head circumference, and body mass index in Turkish children Journal of Clinical Research in Pediatric Endocrinology 2015 7 4 280 293 10.4274/jcrpe.2183 26777039 PMC4805217

[24] Flynn JT Kaelber DC Baker-Smith CM Blowey D Carroll AE Clinical practice guideline for screening and management of high blood pressure in children and adolescents Pediatrics 2017 140 3 e20171904 10.1542/peds.2017-1904 28827377

[25] Bukulmez H Current Understanding of multisystem inflammatory syndrome (MIS-C) following COVID-19 and its distinction from Kawasaki disease Current Rheumatology Reports 2021 23 8 58 10.1007/s11926-021-01028-4 34216296 PMC8254432

[26] Kellum JA Lameire N Group KAGW Diagnosis, evaluation, and management of acute kidney injury: a KDIGO summary (Part 1) Critical Care 2013 17 1 204 10.1186/cc11454 23394211 PMC4057151

[27] Canpolat N COVID-19 and the kidney Turkish Archives of Pediatrics 2021 56 2 97 98 10.5152/TurkArchPediatr.2021.150121 34286316 PMC8269931

[28] Haslak F Varol SE Gunalp A Kaynar O Yildiz M Comparisons of clinical features and outcomes of COVID-19 between patients with pediatric onset inflammatory rheumatic diseases and healthy children Journal of Clinical Medicine 2022 11 8 2102 10.3390/jcm11082102 35456195 PMC9030434

[29] Melgosa M Madrid A Alvárez O Lumbreras J Nieto F SARS-CoV-2 infection in Spanish children with chronic kidney pathologies Pediatric Nephrology 2020 35 8 1521 1524 10.1007/s00467-020-04597-1 32435879 PMC7237873

[30] Marlais M Wlodkowski T Vivarelli M Pape L Tönshoff B The severity of COVID-19 in children on immunosuppressive medication The Lancet Child and Adolescent Health 2020 4 7 e17 e18 10.1016/S2352-4642(20)30145-0 32411815 PMC7220160

[31] Minotti C Tirelli F Barbieri E Giaquinto C Donà D How is immunosuppressive status affecting children and adults in SARS-CoV-2 infection? A systematic review The Journal of Infection 2020 81 1 e61 e66 10.1016/j.jinf.2020.04.026 PMC7179496 32335173

[32] Bush R Johns F Acharya R Upadhyay K Mild COVID-19 in a pediatric renal transplant recipient American Journal of Transplantation 2020 20 10 2942 2945 10.1111/ajt.16003 32406181 PMC7272978

[33] Angeletti A Trivelli A Magnasco A Drovandi S Sanguineri F Risk of COVID-19 in young kidney transplant recipients. Results from a single-center observational study Clinical Transplantation 2020 34 7 e13889 10.1111/ctr.13889 32396985

[34] Columbia University Kidney Transplant Program Early Description of Coronavirus 2019 Disease in Kidney Transplant Recipients in New York Journal of the American Society of Nephrology 2020 31 6 1150 1156 10.1681/ASN.2020030375 32317402 PMC7269361

[35] Fernández-Ruiz M Andrés A Loinaz C Delgado JF López-Medrano F COVID-19 in solid organ transplant recipients: A single-center case series from Spain American Journal of Transplantation 2020 20 7 1849 1858 10.1111/ajt.15929 32301155 PMC9800471

[36] Montagud-Marrahi E Cofan F Torregrosa JV Cucchiari D Ventura-Aguiar P Preliminary data on outcomes of SARS-CoV-2 infection in a Spanish single center cohort of kidney recipients American Journal of Transplantation 2020 20 10 2958 2959 10.1111/ajt.15970 32368838 PMC7267297

[37] Pereira MR Mohan S Cohen DJ Husain SA Dube GK COVID-19 in solid organ transplant recipients: Initial report from the US epicenter American Journal of Transplantation 2020 20 7 1800 1808 10.1111/ajt.15941 32330343 PMC7264777

[38] Akalin E Azzi Y Bartash R Seethamraju H Parides M Covid-19 and Kidney Transplantation The New England Journal of Medicine 2020 382 25 2475 2477 10.1056/NEJMc2011117 32329975 PMC7200055

[39] Nair V Jandovitz N Hirsch JS Nair G Abate M COVID-19 in kidney transplant recipients American Journal of Transplantation 2020 20 7 1819 1825 10.1111/ajt.15967 32351040 PMC7267603

[40] Canpolat N Yıldırım ZY Yıldız N Taşdemir M Göknar N COVID-19 in pediatric patients undergoing chronic dialysis and kidney transplantation European Journal of Pediatrics 2022 181 1 117 123 10.1007/s00431-021-04191-z 34218318 PMC8254625

[41] Haslak F Barut K Durak C Aliyeva A Yildiz M Clinical features and outcomes of 76 patients with COVID-19-related multi-system inflammatory syndrome in children Clinical Rheumatology 2021 40 10 4167 4178 10.1007/s10067-021-05780-x 34089099 PMC8178032

[42] Marlais M Wlodkowski T Al-Akash S Ananin P Bandi VK COVID-19 in children treated with immunosuppressive medication for kidney diseases Archives of Disease in Childhood 2020 106 8 798 801 10.1136/archdischild-2020-320616 33355203 PMC7754669

[43] Stokes EK Zambrano LD Anderson KN Marder EP Raz KM Coronavirus Disease 2019 Case Surveillance - United States, January 22–May 30, 2020 Morbidity and Mortality Weekly Report 2020 69 24 759 765 10.15585/mmwr.mm6924e2 32555134 PMC7302472

[44] Zimmermann P Curtis N Why is COVID-19 less severe in children? A review of the proposed mechanisms underlying the age-related difference in severity of SARS-CoV-2 infections Archives of Disease in Childhood 2020 archdischild: 2020-320338 10.1136/archdischild-2020-320338 33262177

[45] Rajapakse N Dixit D Human and novel coronavirus infections in children: a review Paediatrics and International Child Health 2021 41 1 36 55 10.1080/20469047.2020.1781356 32584199

[46] Cristiani L Mancino E Matera L Nenna R Pierangeli A Will children reveal their secret? The coronavirus dilemma The European Respiratory Journal 2020 55 4 2000749 10.1183/13993003.00749-2020 32241833 PMC7113798

[47] Yang X Tian S Guo H Acute kidney injury and renal replacement therapy in COVID-19 patients: A systematic review and meta-analysis International Immunopharmacology 2021 90 107159 10.1016/j.intimp.2020.107159 33223467 PMC7608016

[48] Fu EL Janse RJ de Jong Y van der Endt VHW Milders J Acute kidney injury and kidney replacement therapy in COVID-19: a systematic review and meta-analysis Clinical Kidney Journal 2020 13 4 550 563 10.1093/ckj/sfaa160 32897278 PMC7467593

[49] Yang X Jin Y Li R Zhang Z Sun R Prevalence and impact of acute renal impairment on COVID-19: a systematic review and meta-analysis Critical Care 2020 24 1 356 10.1186/s13054-020-03065-4 32552872 PMC7300374

[50] Ali H Daoud A Mohamed MM Salim SA Yessayan L Survival rate in acute kidney injury superimposed COVID-19 patients: a systematic review and meta-analysis Renal Failure 2020 42 1 393 397 10.1080/0886022X.2020.1756323 32340507 PMC7241495

[51] Alberici F Delbarba E Manenti C Econimo L Valerio F Management of patients on dialysis and with kidney transplantation during the SARS-CoV-2 (COVID-19) pandemic in Brescia, Italy Kidney International Reports 2020 5 5 580 585 10.1016/j.ekir.2020.04.001 32292866 PMC7128395

[52] Cravedi P Mothi SS Azzi Y Haverly M Farouk SS COVID-19 and kidney transplantation: Results from the TANGO International Transplant Consortium American Journal of Transplantation 2020 20 11 3140 3148 10.1111/ajt.16185 32649791 PMC7405285

[53] Lum E Bunnapradist S Multani A Beaird OE Carlson M Spectrum of Coronavirus disease 2019 outcomes in kidney transplant recipients: A single-center experience Transplantation Proceedings 2020 52 9 2654 2658 10.1016/j.transproceed.2020.09.005 33041077 PMC7832798

[54] Chiotos K Hayes M Kimberlin DW Jones SB James SH Multicenter initial guidance on use of antivirals for children with Coronavirus disease 2019/Severe Acute Respiratory Syndrome Coronavirus 2 Journal of the Pediatric Infectious Diseases Society 2020 9 6 701 715 10.1093/jpids/piaa045 32318706 PMC7188128

[55] Siemieniuk RA Bartoszko JJ Zeraatkar D Kum E Qasim A Drug treatments for COVID-19: living systematic review and network meta-analysis BMJ 2020 370 m2980 10.1136/bmj.m2980 32732190 PMC7390912

[56] Şimşek Yavuz S Ünal S Antiviral treatment of COVID-19 Turkish Journal of Medical Sciences 2020 50 SI-1 611 619 10.3906/sag-2004-145 32293834 PMC7195979

[57] Guner R Hasanoglu I Kayaaslan B Aypak A Akinci E Comparing ICU admission rates of mild/moderate COVID-19 patients treated with hydroxychloroquine, favipiravir, and hydroxychloroquine plus favipiravir Journal of Infection and Public Health 2021 14 10 365 370 10.1016/j.jiph.2020.12.017 33647553 PMC7771901

[58] Kashour Z Riaz M Garbati MA AlDosary O Tlayjeh H Efficacy of chloroquine or hydroxychloroquine in COVID-19 patients: a systematic review and meta-analysis The Journal of Antimicrobial Chemotherapy 2021 76 1 30 42 10.1093/jac/dkaa403 33031488 PMC7665543

[59] Maggiore U Abramowicz D Crespo M Mariat C Mjoen G How should I manage immunosuppression in a kidney transplant patient with COVID-19? An ERA-EDTA DESCARTES expert opinion Nephrology Dialysis Transplantation 2020 35 6 899 904 10.1093/ndt/gfaa130 PMC7313836 32441741

[60] Al Maskari Z Al Blushi A Khamis F Al Tai A Al Salmi I Characteristics of healthcare workers infected with COVID-19: A cross-sectional observational study International Journal of Infectious Diseases 2021 102 32 36 10.1016/j.ijid.2020.10.009 33039607 PMC7543901

